# Spontaneous complete regression of multiple metastases of hepatocellular carcinoma: A case report

**DOI:** 10.3892/ol.2014.1869

**Published:** 2014-02-11

**Authors:** DO HYOUNG LIM, KEON WOO PARK, SOON IL LEE

**Affiliations:** Division of Hematology-Oncology, Department of Medicine, Dankook University College of Medicine, Cheonan 330-715, Republic of Korea

**Keywords:** spontaneous regression, hepatocellular carcinoma, liver cancer, metastasis

## Abstract

Spontaneous regression of cancer is a partial or complete disappearance of malignant tumor without specific treatment. Spontaneous regression of hepatocellular carcinoma (HCC) is a rare condition, and the mechanism underlying it is unclear. This report presents a rare case of spontaneous complete regression of HCC, as revealed by tumor markers and imaging studies. A 64-year-old Korean male patient with hepatitis B virus-associated chronic hepatitis presented with HCC. The patient had undergone right lobectomy of the liver but the cancer recurred with multiple lung and adrenal metastases after 14 months. The patient received palliative cytotoxic chemotherapy. However, there was no clinical benefit and the disease progressed. It was decided to discontinue anticancer therapy and administer only supportive care. After approximately six months, the symptoms disappeared and the HCC had completely regressed. The patient remains alive over five years after recurrence.

## Introduction

Hepatocellular carcinoma (HCC) is the most frequent histological diagnosis of primary liver cancer, the fifth most prevalent type of cancer and the second most common cause of cancer-associated mortality in males worldwide. It is also the seventh most commonly diagnosed cancer and sixth greatest cause of cancer-associated mortality in females worldwide (1). Prognosis for terminal-stage HCC is poor, and the majority of patients succumb to the disease within six months (2). Spontaneous regression of cancer is defined as a partial or complete disappearance of malignant tumors in the absence of specific treatment (3). Spontaneous regression of HCC is a rare condition and the underlying mechanism of this phenomenon is unclear. The present study reports a case of multiple lung, adrenal gland and lymph node metastases of recurrent HCC with spontaneous complete regression, as revealed by tumor markers and imaging studies. The patient provided written informed consent.

## Case report

A 64-year-old male patient was admitted to the Division of Hematology and Oncology, Department of Medicine, Dankook University Hospital (Cheonan, Korea) for treatment of progressive dyspnea and cough. The patient had undergone right lobectomy of the liver due to HCC at Severance Hospital (Seoul, Korea) on October 23, 2006. At that time, the surgical specimen was confirmed to be HCC, Edmondson grade II. After 14 months, a chest computed tomography (CT) scan revealed multiple lung metastases and left adrenal gland metastasis. The level of serum protein induced by vitamin K absence or antagonist II (PIVKA II) was elevated abnormally (207 mAU/ml). Diagnosed with recurrent HCC with multiple lung metastases and adrenal metastasis, the patient received palliative chemotherapy with 5-fluorouracil, adriamycin and carboplatin. Following two cycles of first-line chemotherapy, the disease progressed. The patient was administered two more cycles of second-line chemotherapy with 5-fluorouracil, etoposide and carboplatin, and three cycles of third-line chemotherapy with oral capecitabine. However, the disease demonstrated no objective response and further progressed. The patient and physician agreed to discontinue cytotoxic chemotherapy, and the patient was transferred to Dankook University Hospital for paliative care on February 19, 2009.

**Figure 1:**
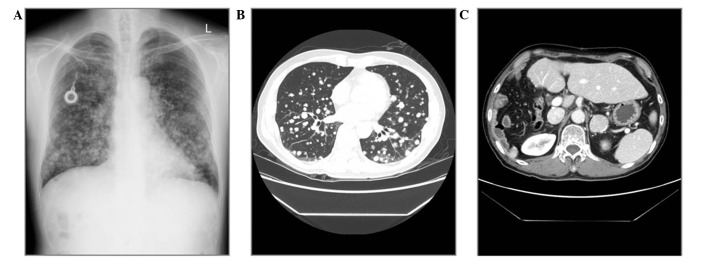
(A) Chest radiography and computed tomography scans of the (B) chest and (C) abdominopelvis in February, 2009 revealed multiple metastatic nodules in the two lung fields, bilateral adrenal metastases and a metastatic lymph node ~1.6 cm in size adjacent to the liver resection margin.

**Figure 2:**
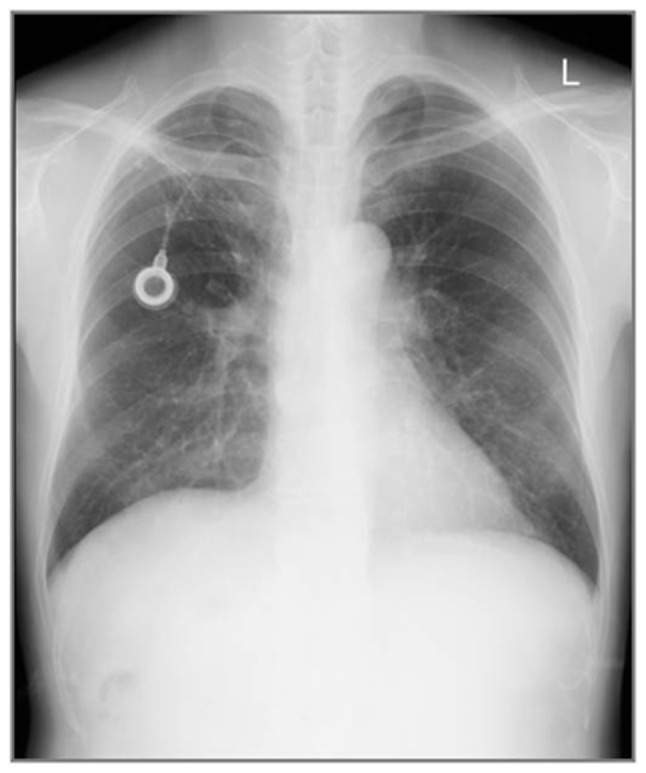
Chest radiograph of the patient in September, 2009 revealed that all metastatic lung nodules had disappeared.

The patient was a chronic hepatitis B virus carrier and an older brother had hepatitis B viral liver cirrhosis. Initial laboratory data from our hospital was as follows: White blood cell count, 4,970 cells/*μ*l; hemoglobin level, 17.2 g/dl; platelet count, 16.4×10^4^ platelets/μl; aspartate aminotransferase : alanine aminotransferase level, 32:41 IU/l; total bilirubin level, 0.97 mg/dl; gamma guanosine triphosphate level, 17 IU/l; alkaline phosphatase level, 89 IU/l; protein : albumin level, 8.4:4.3 g/dl; prothrombin time (international normalized ratio), 13.2 sec (1.14); α-fetoprotein (AFP) level, 16.55 ng/ml (normal range, 0–15 ng/ml); and PIVKA II level, 12,900 mAU/ml (normal range, 0–40 mAU/ml). Hepatitis B virus DNA polymerase chain reaction test results were positive. At the time of admission, a chest radiograph of the patient revealed miliary nodules in the entirety of the lungs. CT scan of the chest and abdominopelvis indicated miliary metastases throughout the lungs, bilateral adrenal metastases and metastatic abdominal lymph nodes (Fig. 1). The patient was observed at regular three-monthly follow-ups. After the first three months, the patient continued to exhibit cough and dyspnea, and there was no definitive interval change of the multiple lung metastases on the chest radiograph. At the second visit on September 14, 2009, the general condition of the patient had improved, and the cough and dyspnea had stopped. A chest radiograph revealed that all metastatic nodules had disappeared (Fig. 2). Serum AFP level had decreased to 1.5 ng/ml. Spontaneous tumor regression of HCC was observed, and whole-body positron emission tomography-CT scan in December, 2009 revealed that all metastatic nodules in the lung, as well as the metastatic lesions of the two adrenal glands and lymph nodes, had disappeared. The patient had taken an alternative herbal medicine for approximately one week, on the recommendation of his family. The herbal medicine was *Dendropanax morbifera* Leveille, which is a subtropical, broad-leaved evergreen tree belonging to the family Araliaceae (4).

**Figure 3:**
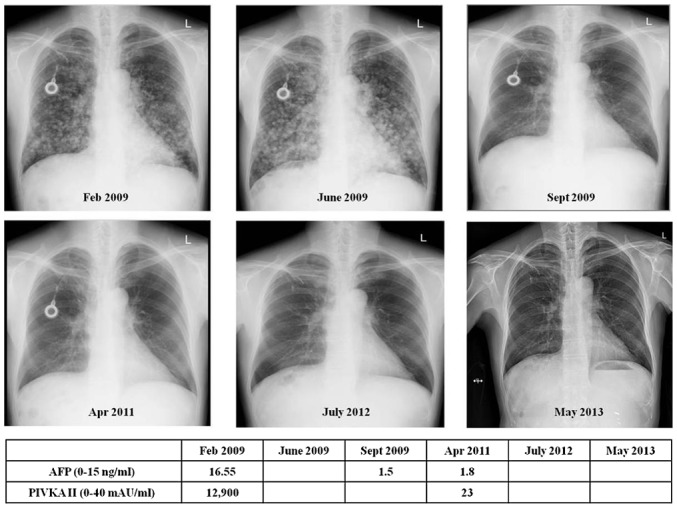
Chest radiograph revealed no metastatic lesions in either lung field from September, 2009. Levels of AFP and PIVKA II were within the normal range. AFP, α-fetoprotein; PIVKA II, protein induced by vitamin K absence or antagonist II.

Follow-up CT scans revealed no recurrent lesions, and follow-up chest radiograph also indicated no metastatic lesions. Serum AFP and PIVKA II levels were within the normal range (Fig. 3). The patient is alive without any symptoms or signs as of May 2013.

## Discussion

The present study reports a rare case of spontaneous complete regression of hepatitis B virus-associated HCC. This phenomenon is consistent with spontaneous regression of cancer as defined by Everson and Cole (3). Spontaneous regression has been described in numerous types of tumors, particularly renal cell carcinoma, melanoma and neuroblastoma. The incidence of spontaneous regression of cancer is difficult to identify, but it has been estimated to occur in 1 in 60,000–100,000 cancer patients (5). Epstein and Leung reported that spontaneous regression of HCC appears to be more frequent than that of other types of cancers (6), and Oquinena *et al* reported that spontaneous regression is not an extraordinary event among patients with HCC (7). Nonetheless, spontaneous complete regression and long-term survival in HCC is extremely rare. In a PubMed search, the present study identified ≥60 reported cases of spontaneous regression of HCC (7–11). Despite several reported cases of spontaneous regression of metastatic HCC, to our knowledge, this is the first case of spontaneous complete regression of a massive HCC with multiple metastatic organs.

No exact mechanism for the spontaneous regression of HCC is known. Several factors have been proposed, including abstinence from alcohol, disruption of tumor blood supply from subintimal vascular injury during angiographic procedures, use of herbal medications, vitamin K, androgens, anti-estrogen therapy, sepsis, fever and blood transfusion (3,45,12–18). Even following detailed examination of all published cases of spontaneous regressing HCC, precise conclusions concerning the mechanisms of spontaneous regression remain unclear.

In the present report, no blood transfusion was performed, and during the follow-up period there was no use of hepatic angiography. There was no history of gastrointestinal bleeding or infection in this patient. As the patient was a social drinker, but had already stopped drinking alcohol at the initial diagnosis of the disease, abstinence from alcohol was not associated with the spontaneous regression.

The patient had taken an alternative herbal medicine: The extracts from *Dendropanax morbifera* Leveille (Korean name: Hwangchil tree), which is a subtropical, broad-leaved evergreen tree belonging to the family Araliaceae. Previous studies have demonstrated that *Dendropanax morbifera* Leveille has anticomplement activity, antiatherogenic activity and antidiabetic effects (19–21). Although the herbal medicine may be associated with regression of HCC, it cannot be concluded that the use of this particular herbal medicine affected the regression of HCC in the present case, as there is no study on the antitumor effects of *Dendropanax morbifera* Leveille, and the patient had taken the herbal medication on only a few occasions during a relatively short time period. The patient received no specific antitumor treatment, nor had any known factors that effect HCC regression. Therefore the regression of HCC presented in this report can be described as truly spontaneous.

In conclusion, spontaneous tumor regression is an interesting phenomenon, and spontaneous regression of HCC is rare but not unknown. Although the mechanism of spontaneous regression of HCC remains unclear and differs from patient to patient, collation and further discussion of such cases will help to advance the understanding of the phenomenon.
